# Anticancer drug discovery from Chinese medicinal herbs

**DOI:** 10.1186/s13020-018-0192-y

**Published:** 2018-07-04

**Authors:** Mu-Yang Huang, Le-Le Zhang, Jian Ding, Jin-Jian Lu

**Affiliations:** 1 State Key Laboratory of Quality Research in Chinese Medicine, Institute of Chinese Medical Sciences, University of Macau, 7014, N22, Avenida da Universidade, Taipa, Macao China; 2 Division of Anti-tumor Pharmacology, State Key Laboratory of Drug Research, Shanghai Institute of Materia Medica, Chinese Academy of Sciences, Shanghai, China

**Keywords:** Chinese medicinal herbs, Anticancer, Drug discovery, Natural products, Formulae

## Abstract

Cancer is still presenting a serious threat to human health worldwide. The understanding of the complex biology of cancer and the development of oncotherapy have led to increasing treatment approaches such as targeted therapy and immunotherapy. Chinese medicinal herbs have attracted considerable attention due to their potential anticancer effects. Some natural products or formulae from Chinese medicinal herbs with directly or indirectly anticancer effects have been reported. In this article, we summarized the current progression on development of anticancer drugs from Chinese medicinal herbs, toward providing ideas for further development and application of Chinese medicinal herbs in cancer therapy.

## Background

Cancer is a high-morbidity and high-mortality disease, which presents a serious threat to human health. According to the global cancer statistics, there was millions of new cancer cases and deaths occur around the world. The statistics also predicted that the number of new cancer cases will constantly increase in the future [1–3]. However, the complexity and covertness of cancer always make it difficult to diagnose at the incipient stage of disease. Cancer often develops into the advanced stage when the patient is diagnosed with neoplastic disease, which extremely augments treatment difficulties. Chemotherapy and radiotherapy are still commonly conventional approaches for treatment of patients harboring advanced cancer [4]. In the past decades, scientists have gained in-depth understanding about the complex biology of cancer [5], which improves the cancer treatment significantly. Recently, targeted therapy and immunotherapy have also been proposed and made a hit [6, 7]. Besides, some complementary or alternative therapies including Chinese medicinal herbs are applied to supplement clinical treatment [8, 9].

Chinese medicinal herbs with multi-target and multi-level function characteristics have earned increasing attention in the fields of cancer prevention and treatment [10]. Studies have shown that some Chinese medicines as well as many classical formulae exhibit prominent anticancer effects and have potentials to supplement cancer treatment [11, 12]. Arsenic trioxide (As_2_O_3_), a toxic Chinese medicines, has been successfully applied in the clinical treatment for patients of acute promyelocytic leukemia [13]. In addition, clinical case reports have presented that some formulae, including PHY906 based on Huang-Qin-Tang (黃芩湯), can improve the life quality of patients and exert synergetic effects with conventional drugs [14]. Chinese medicinal herbs are potential candidates to be developed as new anticancer drugs. In this article, we summarize the current progression of anticancer drug development from Chinese medicinal herbs and discuss some drug discovery strategies, toward providing ideas for their further development and application in the field of cancer therapy.

## Development of natural products

### Natural products with potential anticancer effects

**Fig. 1:**
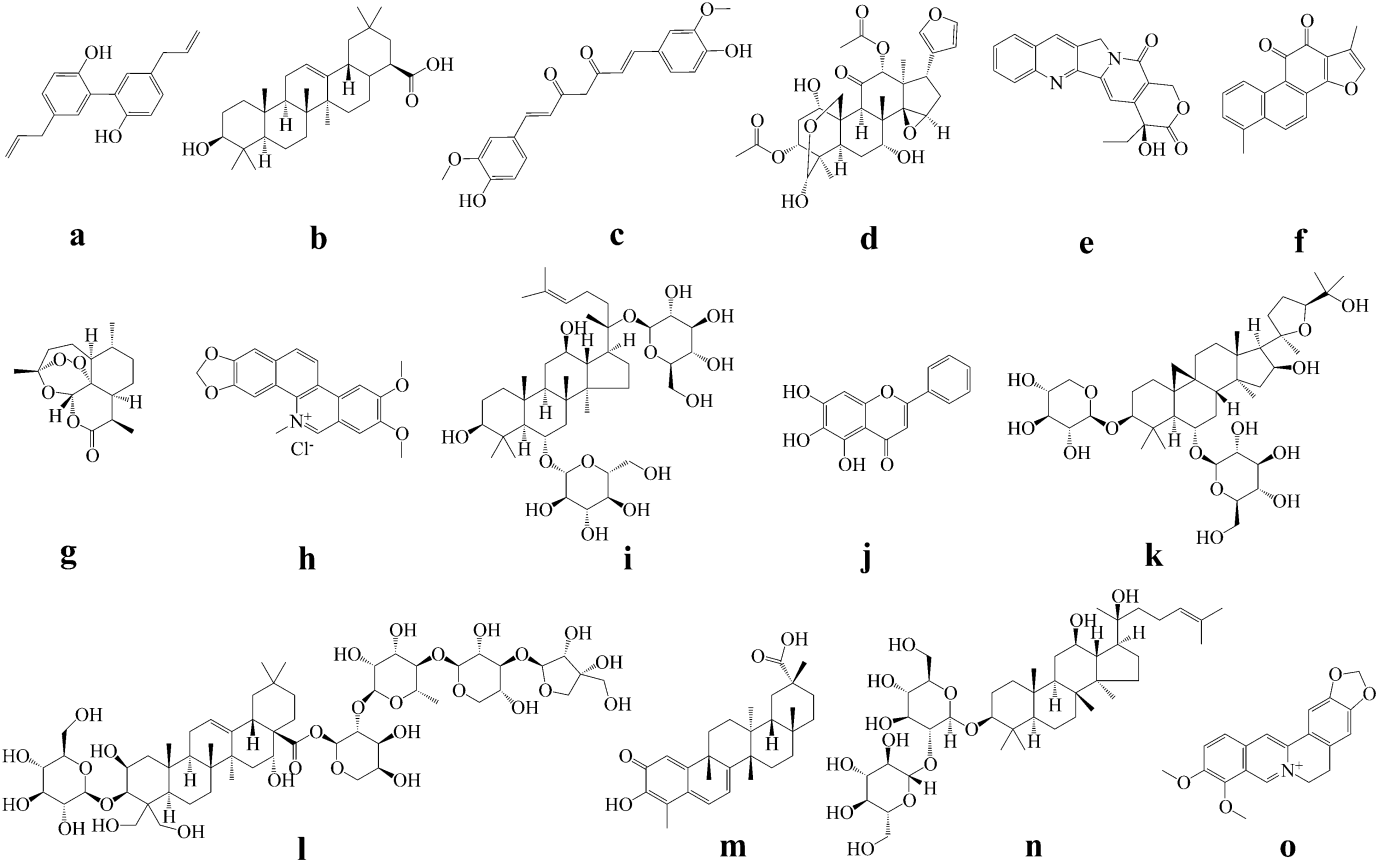
Chemical structures of the natural products. **a** magnolol; **b** oleanolic acid; **c** curcumin; **d** toosendanin; **e** camptothecin; **f** tanshinone I; **g** artemisinin; **h** nitidine chloride; **i** ginsenoside Rg1; **j** baicalein; **k** astragaloside IV; **l** platycodin D; **m** celastrol; **n** ginsenoside Rg3; **o** berberine

Natural products represent a rich source with remarkable chemical diversity for discovery and development of new drugs [15]. The development of modern technologies has broadened the cognition of chemical components of Chinese medicinal herbs. A large number of natural products isolated and identified from Chinese medicinal herbs have been investigated for their anticancer potential [16]. Drug screening is an indispensable step during drug discovery. In consideration of the huge amounts of natural products from Chinese medicinal herbs, preliminary screening using rational and efficient screening models are highly desirable for drug discovery. Over the past decades, some important anticancer agents were discovered based on classical phenotypic screening (function-first), and some were developed from general target-based screening (target-first) [17]. For example, the famous chemotherapeutic drugs including cisplatin and taxanes, were discovered via cytotoxic phenotype investigation and followed by the identification of their mechanisms of molecular actions [18–20]. Some others were developed from target-based discovery, such as epothilone (target tubulin) and temozolomide (target DNA) [21, 22]. Similarly, most of the previous reported natural products with anticancer potential were discovered based on the classical targets screening or the main “criterion” of cytotoxicity profiling. However, few of the natural products reported in the literature has progressed into further clinical evaluation ultimately, leading to the suspect and argument about the rationality and efficiency of these classical models. Along with the better understanding of tumor biology, targeted therapy (BCR-ABL, EGFR, VEGFR, HER2, mTOR, c-Met, etc.) and immunotherapy (CTLA-4, PD-1/PD-L1, etc.) have shown exciting anticancer effects in clinic recently. In this case, we wonder whether natural products isolated from Chinese medicinal herbs can also regulate some important signaling pathways to exert anticancer effects. Magnolol (Fig. 1a), a component isolated from *Magnoliae Officinalis Cortex*, was reported to possess anticancer effects against human breast cancer via inhibiting EGFR signaling pathways [23]. Another study showed that oleanolic acid (Fig. 1b), distributed in many Chinese medicines, exerted inhibition effect against tumor angiogenesis via suppression of STAT3 and Hedgehog pathways [24]. It is well known that various of Chinese medicinal herbs may have potentials to regulate immune system. Discovery of anticancer agents regulating relative immune checkpoints represents another considerable strategy. Recently, researchers have found that curcumin (Fig. 1c), isolated from *Curcumae Longae Rhizoma*, decreased PD-L1 expression and then sensitize the cancer cells to anti-CTLA4 therapy [25]. Those studies suggested that natural products from Chinese medicinal herbs have potentials to regulate some essential signals in cancer cells. Targeting or regulating these proteins/pathways can serve as an accessible drug development strategy, highlighting the need of more specific screening models. Beside the widely used target-based screening, phenotypic screening is another important approach in drug discovery, especially when the specific target or mechanism of action is unclear. With the deeper understanding of cancer biology, more and more phenotypic screening models based on relative disease status such as cell migration, invasion, epithelial-mesenchymal transition (EMT) and stemness, also were established and applied for drug discovery. Tumor cells undergoing EMT exhibit stem-like properties, escape from immune surveillance, and generate resistance to apoptosis and antitumor drugs, and several clinical trials have documented the efficacy of the EMT inhibitory agents as potential anticancer candidates [26]. A study showed that toosendanin (Fig. 1d), isolated from *Toosendan Fructus*, could reverse the transforming growth factor β-induced EMT and morphological change in pancreatic cancer cells and then inhibit the disease progression [27]. Apart from the ‘classic’ cancer phenotypes in vitro, non-cell-autonomous phenotypes such as immune evasion, induction of tumor promoting inflammation and angiogenesis involve cell–cell interactions that are challenging to model and screen in vitro [28]. Taken together, with the increasing discovery of potential therapeutic targets and better understanding of disease process, drug screening platforms remain to be further developed and innovated to obtain lead compounds accurately.

Based on the efficient drug screening, plenty of compounds with potential anticancer effects can be obtained. Lots of natural compounds, including terpenoids, quinones, alkaloids, saponins, coumarins, etc., have been proven to have potential anticancer effects [29–33]. Nevertheless, many natural products which possess superior effects in vitro may not show satisfying results in vivo, owing to their non-specific effect, adverse reactions in vivo, poor solubility or other drug-like properties. Structural modification is a powerful tool to further improve the efficacy of drug candidates and the drug-like properties [34]. For example, camptothecin (Fig. 1e) is a famous natural product isolated from *Camptotheca Acuminata* with good anticancer effects, but its poor solubility and severe side effects lead to many limitations, while its derivatives (structural modification) like topotecan, irinotecan, and belotecan, have been successfully proven in clinic [35, 36]. Recently, study showed that novel nitrogen-enriched derivatives with better anticancer effect and drug-like properties was discovered from structural modification of tanshinone I (Fig. 1f) purified from *Salviae Miltiorrhizae Radix Et Rhizoma* [37]. Beside the anticancer drugs, a series of efficient and diversity oriented artemisinin (Fig. 1g) derivatives with various biological activities have been developed based on the antimalarial agent artemisinin, further proved the importance of structural modification [38, 39]. Furthermore, efforts toward efficient structural modification and total synthesis of bioactive agents have resulted in development of various new synthetic methods such as “Click chemistry”, which may be employed in development of natural products [40].

Beside the chemical structural modification, pharmaceutical method represents another powerful tool to improve medicine safety and drug-like properties. For instance, drug delivery system like antibody–drug conjugate method can enhance therapeutic effects or reduce side effects in some cases. Lidamycin is one of the most potent antitumor agents but causes adverse effects. Antibody–drug conjugate system successfully reduced the side effects caused by lidamycin [41], which set an example for toxic Chinese medicinal herbs development. Other pharmaceutical methods including nanoparticle technology and micelle preparation also have been documented to further improve the solubility and stability of compounds [42]. For example, poor delivery and stability of artemisinin reduce the anticancer effect, but both can be improved by encapsulating artemisinin into nano drug delivery systems. Recently, cucurbit[7]uril, an emerging pharmaceutical excipient was proposed as to improve various properties of drug molecules or active pharmaceutical ingredients. Supramolecular formulation of nitidine chloride (Fig. 1h), a compound from *Zanthoxyli Radix*, by cucurbit[7]uril significantly alleviated its hepatotoxicity and improved its anticancer activity [43]. Totally, based on the preliminary drug screening and evaluation, specific pharmaceutical methods should be taken into consideration in allusion to the characteristics of relative drug candidates.

### Combination therapy

As a complementary or alternative therapy, Chinese medicinal herbs in combination with other drugs serve as a promising strategy [44, 45]. Many investigations on combination therapy have been conducted to overcome the problems that appear during clinical cancer treatment, including side effects, drug resistance, and unsatisfactory treatment outcomes, etc. The strategies of combination therapy which can increase therapeutic efficacy or minimizing toxicity have been highlighted [46], and combination therapy is proposed to be a feasible strategy for Chinese medicinal herbs development.

Adverse drug reactions are common in some conventional cancer treatment, such as chemotherapy and radiotherapy. They may threaten the health of patients and offset the therapeutic benefits of anticancer drugs. Some natural products with effects of anti-inflammation, immune regulation, or toxicity reduction may possess potentials to be developed as adjuvant agents to relieve the side effects during the treatment. Combination therapy with Chinese medicinal herbs has been documented to reduce the toxicity induced by chemotherapy and improve the survival quality of patients [47]. For instance, a study has shown that ginsenoside Rg1 (Fig. 1i), isolated from *Ginseng Radix Et Rhizoma*, can alleviate cisplatin-induced hepatic injury in mice [48]. A number of natural products, such as *Scutellariae Radix* derived baicalein (Fig. 1j) and *Astragali Radix* derived astragaloside IV (Fig. 1k), were demonstrated to relieve the doxorubicin-induced cardiotoxicity [49–51]. Those evidences indicate that side effects induced by the conventional therapy can possibly be alleviated by Chinese medicinal herbs.

Beside alleviating the side effects, combination strategy with Chinese medicinal herbs also exhibit promising potential to improve the therapeutic efficacy, which may be a good choice for some clinical cases wherein patients cannot benefit from monotherapy. Combination of targeted therapy with chemotherapy has been applied in clinic and achieved a good response in some patients [52]. Similarly, natural products isolated from Chinese medicinal herbs also have been demonstrated to exert synergistic effects with other anticancer drugs. Studies have found that platycodin D (Fig. 1l), isolated from *Platycodonis Radix*, remarkably enhanced the antiproliferative effects of AKT and mTOR inhibitors [53, 54]. The anticancer efficacy of TRAIL/APO-2L was dramatically improved by celastrol (Fig. 1m), a natural product purified from *Tripterygium wilfordii*, both in vitro and in vivo [55]. Combination of cisplatin with dihydroartemisinin, a derivative of artemisinin, significantly decreased tumor size in lung carcinoma xenografted mice model via downregulation of VEGFR [56]. Synergistic effect of Dan-Shen-Injection (丹參注射液) was reported when combined with chemotherapy or antiangiogenic therapy [57]. Therefore, combination therapy with Chinese medicinal herbs is a good strategy for enhancing the anticancer effects. As mentioned above, although immunotherapy has achieved great success, only a minority of patients could respond to PD-1/PD-L1 antibodies. We hypothesize that some Chinese medicinal herbs with immunoregulation effects may sensitize patients to immunotherapy.

Additionally, identification of natural products to overcome drug resistance represents another important strategy since drug resistance lead to treatment failure in clinic and remains an obstacle in cancer therapy [58]. Aside from discovering new alternative drugs, scientists have also devoted to find combination strategies to overcome drug resistance. It is reported that various types of mechanisms such as overexpression of some transporters, activation of bypass signaling, and emergence of EMT would lead to drug resistance during cancer treatment [59, 60]. One strategy is to screen natural products that can potentially prevent or delay resistance of tumor to relative drugs. A study has shown that ginsenoside Rg3 (Fig. 1n), purified from *Ginseng Radix Et Rhizoma*, provides a new regimen to delay acquired resistance of EGFR tyrosine kinase inhibitors and ultimately improves median progression-free survival and overall response rate in advanced non-small cell lung cancer patients [61]. For the patients with acquired resistance, overcoming the resistant status or re-sensitizing the therapeutic effects serve as another strategy. A study suggested that *Coptidis Rhizoma* derived berberine (Fig. 1o), which has been widely used in intestinal infection treatment, sensitized the therapeutic effect of cisplatin in the cisplatin-resistant ovarian cancer cells through the miR-93/PTEN/AKT signaling pathway [62].

## Development of Chinese medicinal formulae

Formulae are the main forms of Chinese medicines used in clinic, which also play an essential role in cancer therapy. Numerous formulae have been recorded, but the working mechanisms for most of them remain unknown. Clinically, they are often used in combination with conventional drugs to achieve a synergistic anticancer effect or to reduce the side effects. For example, the formula Xue-Fu-Zhu-Yu-Tang (血府逐瘀湯) decreased the tumor weight by enhancing the immune function in the tumor-bearing mice, and the combinational use of Xue-Fu-Zhu-Yu-Tang increased the effect of chemotherapy [63, 64]. The Sheng-Mai-Yin (生脈飲) can relieve the myocardial toxicity caused by doxorubicin [65]. However, the difficulties of quality control (QC) and mechanism study still represent the main obstacles for the development and clinical usage of Chinese medicinal formulae.

QC is a critical step in Chinese medicinal herbs’ development, since the quality standard is essential to ensure the safety and efficacy. Recently, technologies for the QC of Chinese medicinal herbs have made great progress, while many problems, such as single QC markers and lack of biological effect evaluation, still need to be improved. Active ingredient or high-content component-based QC is unitary and lack of association with pharmaceutical/biological effects, which make it difficult to evaluate the comprehensive quality of complicated formulae. In this case, a more comprehensive QC pattern is required to guarantee the reproducibility and efficacy in formulae development. PHY906, an adjuvant based on a 1800 year-old formula called Huang-Qin-Tang showed potential to increase the therapeutic index of cancer treatment in several studies [66–68]. The development of PHY906 gave a paradigm of comprehensive QC pattern in formulae exploitation, indicating that both chemical analysis and pharmacology/biology evaluation are important. For chemical analysis, more and more efficient and accurate approaches like chromatographic fingerprint technique are used to analyze the components in Chinese medicines. For instance, 162 compounds in Ji-Tong-Ning-Tablet (脊痛寧片) were detected via an UPLC-Q–TOF–MS method, which significantly improved its QC [69]. Besides, pharmacokinetic metabolites also play essential roles in the prediction and management of therapeutic effects, which can be detected by serum pharmacochemistry methods or metabonomics methods. Study showed that 22 absorbed prototypes and 16 metabolites of Da-Bu-Yin-Wan (大補陰丸) were successfully analyzed from rats’ serum after the administration of Da-Bu-Yin-Wan [70]. Apart from chemical analysis, the pharmacological or biological effects should be taken into consideration for QC. Appropriate biological assays based on pharmacological or biological effects and mechanisms of the formulae might improve their QC [71]. In total, to ensure the reliability and uniformity of Chinese medicinal formulae in anticancer drug development and clinical usage, both chemical analysis and pharmacology/biology evaluation based comprehensive patterns of QC need to be further developed.

Mechanism study is another bottleneck for the development of Chinese medicinal formulae, due to complicated components, multi-target effects, complicated interaction with organisms, etc. The traditional ‘one target, one drug’ mechanism study mode is unsuitable for the development of formulae. Along with the application of multi-omics, systems biology and network pharmacology have been developed rapidly and offer promising potential in the mechanism study of formulae. Realgar-Indigo naturalis (複方磺黛片) is an effective formula used for the treatment of acute promyelocytic leukemia. The systems biology strategy based on the molecular, cellular, and organism levels evaluation in vitro and in vivo has been successfully used in its mechanism study, which demonstrated that Realgar-Indigo naturalis formula intensified degradation of promyelocytic leukemia-retinoic acid receptor alpha oncoprotein, increased reprogramming of myeloid differentiation regulators, and enhanced G0/G1 arrest in acute promyelocytic leukemia cells [72]. Similarly, the network pharmacology approach was also successfully applied to clarify the possible therapeutic mechanisms of Liu-Wei-Di-Huang-Wan (六味地黃丸). The results indicated that the effects of Liu-Wei-Di-Huang-Wan on the “Yin deficiency” pattern in Chinese medicine was mediated by maintaining homeostasis in the endocrine system, the immune system and metabolism [73]. Recently, a concept of integrated pharmacology was proposed and used in the mechanism study of formulae, which integrated many subjects such as chemistry, pharmacokinetics, pharmacology, computational science and so on. Based on the integrated pharmacology, the multi-target and multi-component mechanism of Wen-Dan-Tang (溫膽湯), a Chinese herbal formula for treatment of metabolic syndrome, has been thoroughly studied. Six peroxisome proliferators-activated receptors were predicted as targets that exhibited strong binding affinities with 217 active ingredients of Wen-Dan-Tang [74]. Taken together, these approaches provide new insights into the mechanism study of anticancer formulae as well as further discovery of anticancer drugs from Chinese medicinal herbs.

## Discussion

**Fig. 2:**
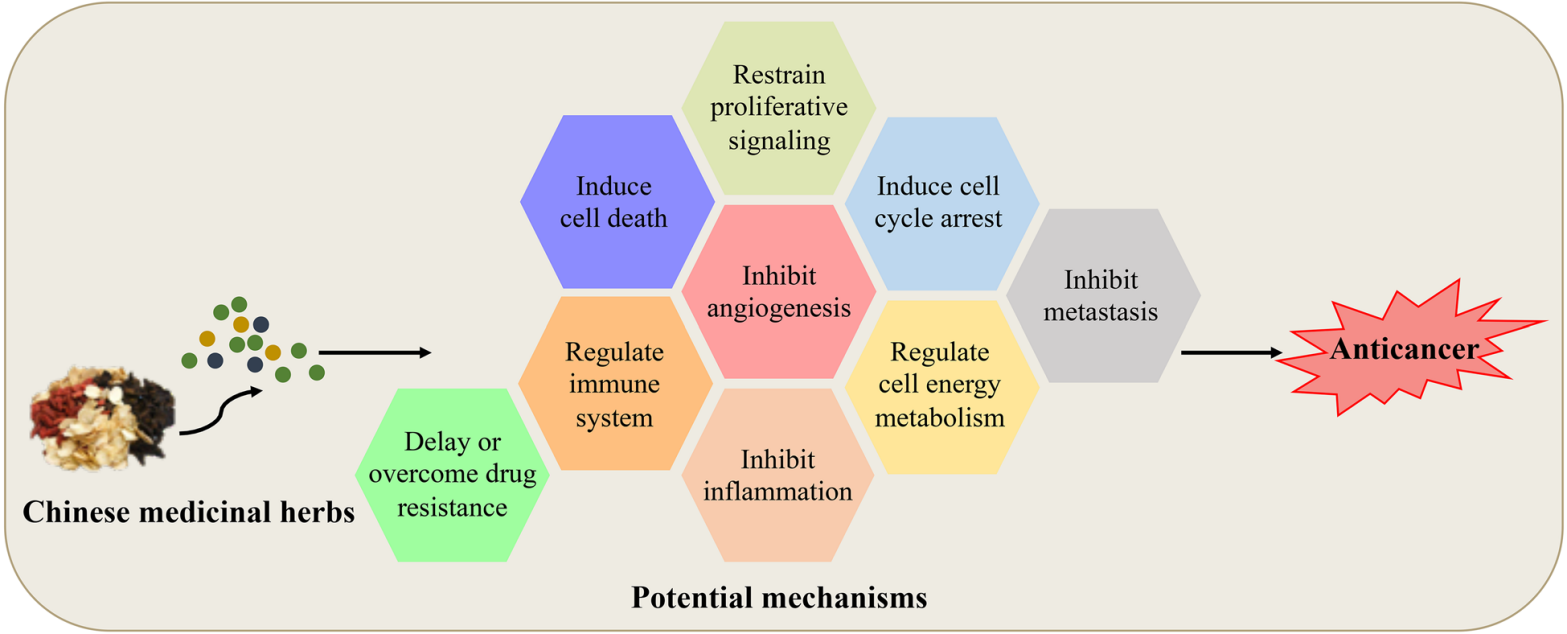
Potential anticancer mechanisms of Chinese medicinal herbs

**Fig. 3:**
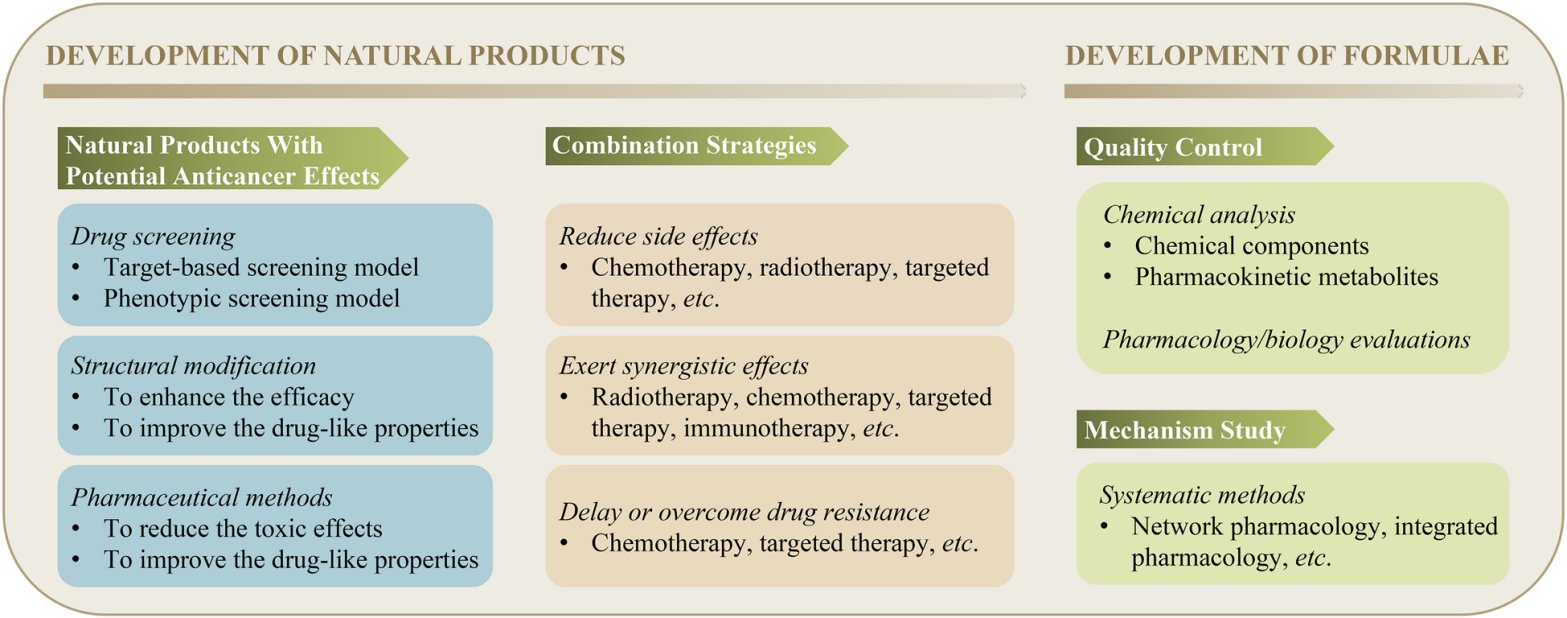
Anticancer drug development strategies from Chinese medicinal herbs

To date, a number of Chinese medicinal herbs (both natural products and formulae) have been documented to possess anticancer activities through various potential mechanisms (Fig. 2). Meanwhile, some of the Chinese medicinal herbs can improve the therapeutic outcomes of patients when used in combination with conventional anticancer drugs due to the presence of synergetic effects, alleviation of side effects, or delaying/overcoming of drug resistance. This manuscript summarizes the anticancer drug development strategies from Chinese medicinal herbs (Fig. 3). Although abundant work about Chinese medicinal herbs have been carried out in the past decades, several points still need to be taken into consideration for future development. Firstly, an effective QC is crucial to ensure the safety and efficacy of Chinese medicinal herbs, especially for formulae. Since numerous factors influence the effects of Chinese medicinal herbs, a more comprehensive QC pattern is required. Aside from some rising chemical analysis methods, such as chromatographic fingerprint and multi-component quantification [75, 76], pharmacology/biology evaluation is needed. Secondly, although Chinese medicinal herbs and formulae have been widely used in clinic, especially in China, the working mechanisms for most of them still remain to be clarified. The better understanding of the involved mechanisms will promote the discovery of more potential anticancer compounds or formulae. Thirdly, many natural products have been extensively studied and proven to exhibit anticancer effects in vitro, while showing poor activity in vivo. Such limitations may be caused by their poor bioavailability or toxicity. Thus, the implementation of some chemical and pharmaceutical methods is necessary during drug development. For those which have been proven safe and effective in vitro and in vivo, clinical trials can be considered under a good quality control. Fourthly, previous investigations mainly focused on the natural products which show high contents in Chinese medicinal herbs owing to the limitation of chemical analysis and isolation technologies. However, beside those high-content compounds, there are still many low-content components existing in Chinese medicinal herbs, which also presents a huge resource for drug development. With the development of more and more advanced chemical analysis technologies and screening models, those low-content compounds can be purified and identified for drug discovery. Fifthly, the microbiota of healthy human is in a state of dynamic equilibrium, and a recent study indicated that the imbalance of microbiota would be associated with various types of diseases including cancer [77]. Microbiota can not only promote the occurrence and development of tumor, but also exhibit inhibitory effect against tumor development in some cases [78, 79]. Meanwhile, it was reported that microbiota was associated with the effects of immunotherapy such as anti-CTLA4 and anti-PD-L1 therapy [80, 81]. We wonder whether the anticancer effects of some Chinese medicinal herbs are related with the regulation of microbiota. Last but not least, in recent years, artificial intelligence technology has been introduced into the field of drug discovery and applied in almost all aspects of drug development, such as drug screening and target predicting [82]. Considering the complexity of Chinese medicinal herbs, application of artificial intelligence technology may promote the development of anticancer drugs from Chinese medicinal herbs.

## Conclusion

Chinese medicinal herbs provide abundant resource library for drug development. It is extensively potential to discover more anticancer drugs from both natural products and traditional formulae. In this review, we documented the current progression on development of anticancer drugs from Chinese medicinal herbs including the natural products and formulae, along with the defects and obstacles remain to be overcome. A series of drug development strategies and technical approaches that suitable for discovery and development of anticancer drugs from Chinese medicinal herbs have been summarized and discussed. Totally, anticancer drug discovery from Chinese medicinal herbs still need a lot of hard work.

## Abbreviations

As_2_O_3_: arsenic trioxide
EGFR: epidermal growth factor receptor
VEGFR: vascular endothelial growth factor receptor
HER2: human epidermal growth factor 2
mTOR: mechanistic target of rapamycin kinase
PD-1/PD-L1: programmed cell death 1/programmed cell death ligand 1
CTLA-4: cytotoxic T-lymphocyte associated antigen 4
EMT: epithelial-mesenchymal transition
TRAIL/APO-2L: tumor necrosis factor α (TNF-α)–related apoptosis-inducing ligand
QC: quality control
UPLC-Q–TOF–MS: ultra-performance liquid chromatography combined with quadrupole time of flight mass spectrometry
